# Protective Effect of *Escherichia coli Nissle 1917* on *Salmonella typhimurium* Infection by Regulating Intestinal Flora

**DOI:** 10.3390/microorganisms14051155

**Published:** 2026-05-20

**Authors:** Zi-Jun Li, Ling-Jiao Yu, Ya-Xin Yang, Ying Li, Emad Beshir Ata, Yang Zhou, Rong-Rong Zhang, Yi-Bing Lian, Hong-Liang Chen, Chun-Wei Shi, Gui-Lian Yang, Hai-Bin Huang, Yan-Long Jiang, Jian-Zhong Wang, Xin Cao, Nan Wang, Yan Zeng, Wen-Tao Yang, Chun-Feng Wang

**Affiliations:** 1 College of Veterinary Medicine, College of Animal Science and Technology, Jilin Provincial Engineering Research Center of Animal Probiotics, Key Laboratory of Animal Production and Product Quality Safety of Ministry of Education, Jilin Agricultural University, Changchun 130117, China; lizijun0623@163.com (Z.-J.L.); YLJ13351513786@163.com (L.-J.Y.); 18602684136@163.com (Y.-X.Y.); zhouyang19970516@163.com (Y.Z.); 18147505147@163.com (R.-R.Z.); 15844888334@163.com (Y.-B.L.); chenhongliang@jlau.edu.cn (H.-L.C.); shichunwei@jlau.edu.cn (C.-W.S.); yangguilian@jlau.edu.cn (G.-L.Y.); huanghaibin@jlau.edu.cn (H.-B.H.); yanlong_jiang@126.com (Y.-L.J.); wangjianzhong@jlau.edu.cn (J.-Z.W.); caoxin@jlau.edu.cn (X.C.); wangnan@jlau.edu.cn (N.W.); zengyan@jlau.edu.cn (Y.Z.); yangwentao@jlau.edu.cn (W.-T.Y.); 2 College of Food and Pharmaceutical Engineering, Wuzhou University, Wuzhou 543002, China; 3 Parasitology and Animal Diseases Department, Veterinary Research Institute, National Research Centre, Dokki, Cairo 12622, Egypt; emadvet2003@yahoo.com

**Keywords:** Nissle 1917, *Salmonella*, probiotics, gut microbiota protection, challenge

## Abstract

Salmonellosis is a global foodborne pathogen with zoonotic importance that seriously threatens livestock breeding and human health. Due to the implementation of an anti-resistance policy, probiotics as an alternative to antibiotics have attracted widespread attention. In this study, the widely used probiotic *Escherichia coli Nissle* 1917 (EcN) was selected to study its protective effect on mice infected with *Salmonella typhimurium*. Two mice groups (*n* = 15) were treated with either EcN and PBS. Flow cytometry showed that the frequency of mature dendritic cells in the Peyer’s patch was significantly increased compared to the PBS group. Previous administration of EcN protected against challenge with *Salmonella typhimurium* infection as an increased survival rate of the mice, a decreased degree of pathological changes, and the number of live bacteria in the spleen and liver were recorded compared to the control group. The results of 16S rRNA high-throughput sequencing of fecal microbial flora showed that EcN could reduce the abundance of microorganisms in the intestine and reduce the proportion of *Lactobacillus*, while *Ruminococcaceae* sp., *Rikenella* sp. and *Bifidobacterium* sp. disappeared. In contrast, the abundance of *Bacteroides* increased, which reduced the effect of *Salmonella typhimurium* on the distribution of intestinal microorganisms. Our results demonstrated that EcN has a protective effect against *Salmonella typhimurium* infection and may act as a candidate probiotic bacterium to apply in the future.

## 1. Introduction

Animals play an important role in maintaining global food security [1,2,3]. They were subjected to different pathogens that affect their productivity [4,5,6]. They have a wide host range and can be transmitted in multiple ways. Food is the most common source of infection, which poses a major threat to livestock, poultry health, and human public safety [7]. *Salmonella* sp. is a significant foodborne pathogen that can cause salmonellosis in humans, cattle, and pigs, and also causes human typhoid fever-like illness in mice [8]. It was discriminated as the main cause of food poisoning in many countries. According to WHO statistics, approximately 16 million people are infected with this bacterium every year, resulting in 600,000 deaths.

The standard treatment for bacterial infection including salmonellosis is still primarily antibiotics [9], but with the prevalence of highly resistant strains and the implementation of a ban on feed antibiotics, we must use a new means to control *Salmonella* infection [10]. Probiotics are living biological agents that have a beneficial effect on recipients at an appropriate range of concentrations [11]. They mainly protect against diseases and stimulate the production of IgA by upregulating the expression of related proteins in the host intestine [12]. To maintain their viability, probiotics must colonize the gut in acidic environments of the stomach and bile [13].

Many studies have shown that probiotics can not only slow *Salmonella* sp. infection [14], but also might be able to inhibit *Salmonella* sp. infection by improving the intestinal flora of diseased animals, increasing the concentration of IgA, and enhancing the defense ability of the gastrointestinal tract [15].

*Escherichia coli* Nissle 1917 (EcN) is one of the few Gram-negative bacteria among probiotics. It is mainly used for the clinical treatment of gastrointestinal dysfunction, such as Crohn’s disease and ulcerative colitis [16]. EcN can directly antagonize intestinal bacteria by producing two kinds of microcin and by sending molecules to the host epithelial tissue to indirectly antagonize the invasion of harmful bacteria [17].

As the bridge between innate and acquired immunity, dendritic cells are the most powerful antigen-presenting cells [18,19]. Previous studies have shown that EcN can promote the maturation of these dendritic cells. Thus, the immune response is enhanced by EcN in vivo, and EcN can stimulate productions of the high levels of cytokines, which play a key role in the formation of innate and acquired immune responses. Therefore, dendritic cell maturation and cytokine production direct the immune response [20]. Changes in dendritic cells in the lymph nodes of mice treated with EcN were detected by flow cytometry. The results showed that EcN increased the proportion of mature dendritic cells and enhanced the immune response.

Enteric *Salmonella* infection poses a major threat to both global public health and livestock industries, causing substantial economic losses. Although the probiotic *Escherichia coli* Nissle 1917 (EcN) demonstrates a potential capacity for preventing bacterial infections, its specific protective mechanisms against intestinal *Salmonella* sp., particularly how it mediates colonization resistance through the modulation of gut microbiota structure, remain to be systematically elucidated. In this study, mice challenged with *Salmonella typhimurium* were protected by EcN. The results suggested that EcN could ameliorate liver pathological damage, lower mortality, reduce the levels of *Salmonella* in the spleen or liver, and then regulate the gut microbes by regulating the distribution of intestinal microflora.

## 2. Materials and Methods

### 2.1. Bacteria

The Gram-negative probiotic strain *E. coli* Nissle 1917 (EcN) was kindly provided by Yong Loo Lin School of Medicine, National University of Singapore (Singapore) [21]. *Salmonella typhimurium* (ATCC14028) was kindly provided by Jilin Provincial Engineering Research Center of Animal Probiotics, Jilin Agricultural University (Changchun, China) [22].

### 2.2. Animals

The female C57BL/6 mice (6 weeks of age) which were specific pathogen-free were obtained from the Beijing HFK Bioscience Co., Ltd., Beijing, China. The mice were allowed to freely drink and eat. The animals were not exposed to any antibiotics.

### 2.3. Salmonella sp. Gavage and Infection in Mice

**Figure 1:**
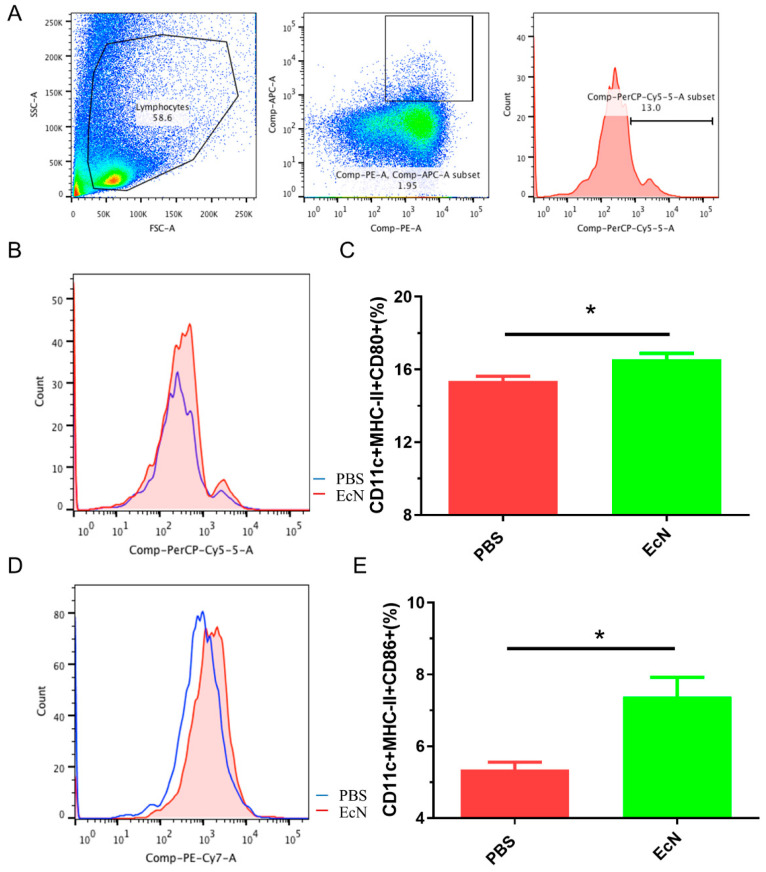
The frequencies of the DC costimulatory molecules CD80 and CD86 on the surface of the Peyer’s patch were evaluated by flow cytometry analysis. (**A**) Strategy of gating, (**B**) counts of CD80^+^, (**C**) *t*-test analysis of CD80^+^ between two groups, (**D**) counts of CD86^+^, (**E**) *t*-test analysis of CD86^+^ between two groups. PBS group, fed PBS, EcN group, fed EcN (n = 3 per group) (* *p* < 0.05).

To study the immune effect of *E. coli Nissle 1917* on mice infected with *Salmonella typhimurium*, we randomly selected 30 six-week-old SPF C57BL/6 mice and divided them into two groups (*n* = 15). The experimental group was given *E. coli Nissle 1917* at a dose of (5 × 10^8^ CFU) orally for 7 days at 200 μL/per mouse; meanwhile, the control group was given PBS orally for 7 days, at 200 μL/per mouse. Eight days later, challenge of the mice in these two groups with *Salmonella typhimurium* at 200 μL/per mouse (2 × 10^8^ CFU) was performed. The body weight and physiological state of the mice were recorded daily across the whole experiment. The spleens and livers of the mice were collected aseptically for bacteria loading determination and histological observation (Figure 1). The cecal contents representing fecal samples were collected and stored at −80 °C for DNA extraction and sequencing.

### 2.4. Flow Cytometric Analysis

The cell suspension made from the Peyer’s patch of mice was washed with phosphate-buffered saline (PBS) and stained with APC-labeled anti-mouse CD11c, PE-labeled anti-mouse MHC-II, PerCP-Cyanine5.5-labeled anti-mouse CD80, and PE-Cyanine7-labeled anti-mouse CD86 (eBioscience, San Diego, CA, USA) at 4 °C for 20 min in the dark [23]. A total of 10,000 live cells were analyzed by flow cytometry (BDFACS LSR Fortessa™, Franklin Lake, NJ, USA). The gate strategy is shown in Figure 1. All flow cytometric data were analyzed using FlowJo 7.6.1 software.

### 2.5. Histopathological Analysis

On the 5th day after challenge, 5 mice in each group were randomly euthanized, and the liver, cecum, and spleen were obtained, fixed with 4% formaldehyde for 48 h, and then dehydrated in an ethanol gradient. Then, the tissue was made transparent with xylene. Then, the tissue was sealed in wax. Tissue embedding and patching were performed after wax immersion. The cubes were sliced at a thickness of 3 μM and then cooled to room temperature for HE is staining [24,25]. Finally, the film was sealed with gum. The cells were observed under a microscope after drying at room temperature.

### 2.6. Colony Count

Five days after challenge, five mice in each group were euthanized, and the livers were collected and placed in an EP tube. After the samples were weighed, sterilized PBS buffer was added at a dose of 1 mL/g. After full grinding and mixing, the liver was diluted to appropriate multiple aliquots. The diluted grinding solution (100 μL) was dropped onto the SS culture dish and cultured at 37 °C for 24 h. After culture, the number of viable *Salmonella typhimurium* in each gram of tissue was counted.

### 2.7. High-Throughput Sequencing of 16S rRNA

Fecal samples were processed according to a previously published method. DNA was extracted from the fecal samples using the QIAamp^®^ DNA Fecal Mini Kit (Qiagen, Shanghai, China, Cat. No. 515 04), and the DNA concentration was determined. The V3–V4 hypervariable region of 16S rRNA was amplified by a pair of the primer pair 341f (5′-CCTACGGGNGGCWGCAG) and 805r (5′-GACTACHVGGGTATCTAATCC′). The sequencing platform used is the Illumina MiSeq platform. To assess sample complexity, normalization was performed according to the minimum sequencing depth across all samples, enabling subsequent alpha and beta diversity analyses. Alpha diversity was quantified using the dominance index calculated via QIIME2 (v1.7.0). For beta diversity visualization, dimensionality reduction was achieved through principal coordinate analysis (PCA), with graphical outputs generated using the ggplot2 package (v4.0.3) [26]. The specific procedures were as follows: pre-denaturation at 94 °C for 3 min, denaturation at 94 °C for 30 s, annealing at 50 °C for 30 s, extension at 72 °C for 60 s, 25 cycles, and finally extension at 72 °C for 7 min. The polymerase chain reaction was repeated three times, and the products were finally purified [27,28]. Sequencing was performed by Novogene (Beijing) Bioinformatics Technology Co., Ltd. (Beijing, China) on the Illumina NovaSeq6000 platform. Raw sequencing data have been deposited to the NCBI Sequence Read Archive (SRA) database under BioProject accession number PRJNA1419175.

### 2.8. Bioinformatics Analysis

By analyzing the original readings of the sequenced samples, α diversity and β diversity indices were assessed. The Venn diagram counted the common and unique OTUs between these samples and revealed the OTUs and the overlap between these groups visually. Additionally, microbial differences between different groups were measured by LEFSE. By calculating the LDA score, and constructing the classification branching map, we can observe the variance of microbial composition. LEfSe was defined as significance less than 0.05 and LDA effect greater than 3. Based on the specified classification unit of the OTU, we detected the relative richness of functional categories. In order to interpret 16S amplification sequence data by a biological perspective, PICRUST was designed [18,29]. The raw reads were deposited into the NCBI Sequence Read Archive database (accession: SRP328586).

### 2.9. Statistical Analysis

The results were presented as the means ± SEM and significance was assessed using Student’s *t*-test. A *p* value < 0.05 was considered significant.

## 3. Results

### 3.1. EcN Can Increase the Frequency of Activated Dendritic Cells in Mice

The frequency of mature dendritic cells was detected by flow cytometry in both the EcN and PBS groups. Dendritic cells were screened by CD11c^+^ MHC-II^+^ (Figure 1A). CD80 and CD86 were expressed on the surface of dendritic cells. By comparing the proportion of CD80 (Figure 1B) and CD86 (Figure 1C) expressed in dendritic cells, an increased level of the mature dendritic cells of the mice treated with EcN (*p* < 0.05) was determined comparing to the PBS control group, indicating that EcN could enhance the immune response in vivo.

### 3.2. EcN Significantly Protected Mouse Challenged with Salmonella typhimurium Infection

**Figure 2:**
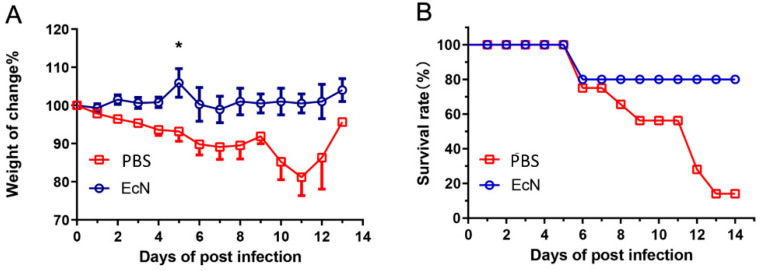
*E. coli Nissle 1917* reduced weight loss, death and cecal injury caused by *Salmonella*. (**A**) Weight lose rate, (**B**) survival rate. PBS group, PBS provided and treated with *Salmonella*, EcN group, EcN provided and treated with *Salmonella* (n = 15 per group) (* *p* < 0.05).

The mouse weight and physiological status were used as evaluation parameters. The obtained results clarified that, after challenging with *Salmonella typhimurium*, the mouse weight in the EcN group was almost unchanged; meanwhile, that in the PBS group was significantly decreased. Similarly, the mouse status in the PBS control group had a poor appetite before death, although there was no significant change in the EcN group (Figure 2A). Furthermore, The EcN had a protective effect against *Salmonella* infection in mice as the EcN mice group showed a higher survival rate after challenging with a complete protection, although the mice in the PBS control group began to die on day 6. Furthermore, a value of 10% of final survival rate was observed on day 14 (Figure 2B). The experiment showed that EcN had a protective effect on mice challenged with bacteria.

### 3.3. EcN Mitigates Histopathological Variations in Mice with Infection

**Figure 3:**
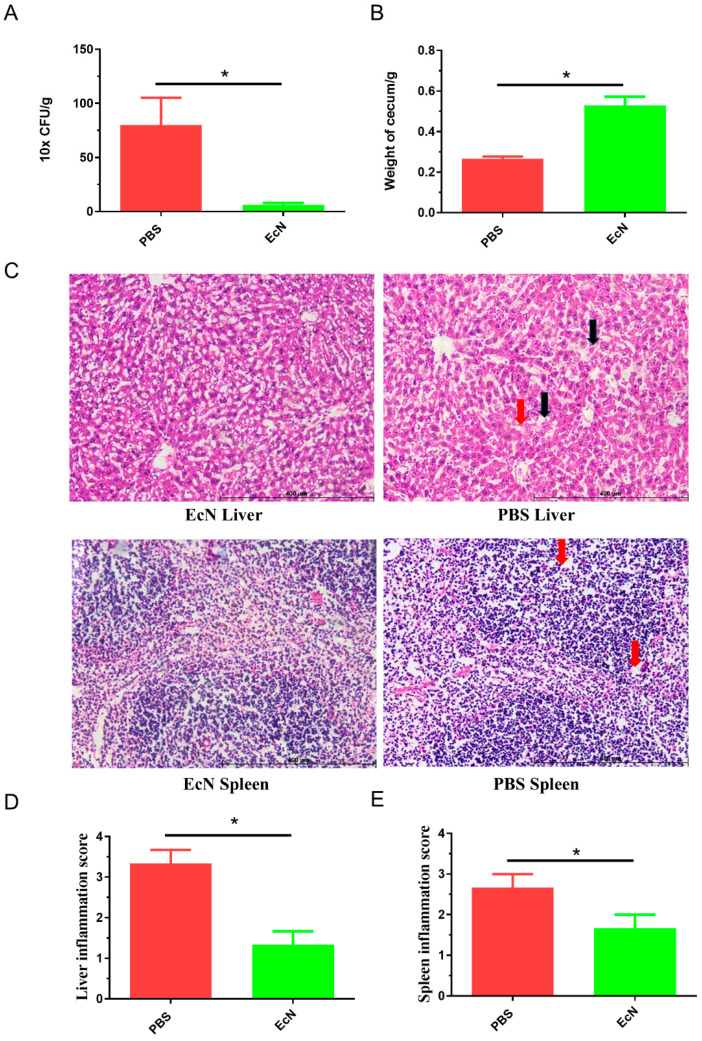
*E. coli* Nissle 1917 reduced the damage and the amounts of live bacteria in the liver and spleen after *Salmonella* infection. (**A**) Live bacteria in the liver. (**B**) Weight of cecum. (**C**) Histological observation. Tissue edema marked with black arrows, and blurred edges marked with red arrows. (**D**) Liver inflammation score. (**E**) Spleen inflammation score. Group EcN: EcN provided and treated with *Salmonella*, Group PBS, PBS provided and treated with *Salmonella* (scale bars: 400 μm) (n = 5 per group) (* *p* < 0.05).

The postmortem examination of the euthanized mice revealed that the cecum of the mice from the PBS group had a bleeding content and was significantly smaller than that of the mice in the EcN group. Also, the weight of the cecum in the EcN group was significantly higher than the control PBS group (Figure 3A). This preliminary finding indicated the protective intestinal tract of mice administered with EcN.

Hemorrhage spots and gray-white necrotic lesions were observed in the livers of the mice in the PBS group. Microscopic examination showed focal necrosis of hepatocytes. The hepatocytes in the necrotic lesions showed no structural red staining, bleeding or inflammatory cell infiltration. On the other hand, microscopic examination of the mice in the EcN group showed slight bleeding, and the lesion degree was significantly reduced compared with that in the control group. This experiment proved that EcN could reduce the damage to the liver tissue of mice infected with *Salmonella*.

The spleen of the PBS group was significantly larger than that of the EcN group during autopsy. Histopathologically, the marginal tissue of spleen cells in the PBS group was vague and incomplete with slight edema. On the other hand, spleen cells and tissues of mice in the EcN group were intact without obvious hyperemic swelling. This experiment showed that EcN could reduce spleen damage in mice infected with *Salmonella typhimurium* (Figure 3B).

### 3.4. EcN Decreases the Amounts of Salmonella typhimurium in the Liver

By counting the number of live *Salmonella* in the liver of mice infected with *Salmonella typhimurium*, we found that no or a low count number of *Salmonella typhimurium* was found in the liver of the mice in the EcN group, while the number of *Salmonella typhimurium* in the liver of mice in the infection group was larger than 75 CFU/g, meaning 12.3 times larger than that of the mice in the EcN group (*p* < 0.05). This experiment indicated that EcN could decrease the number of live bacteria in the livers of mice infected with *Salmonella typhimurium* (Figure 3C).

### 3.5. Alpha and Beta Diversity Indices of the Gut Microbial Community in Mice with Salmonella typhimurium Infection

#### 3.5.1. Alpha Diversity

**Figure 4:**
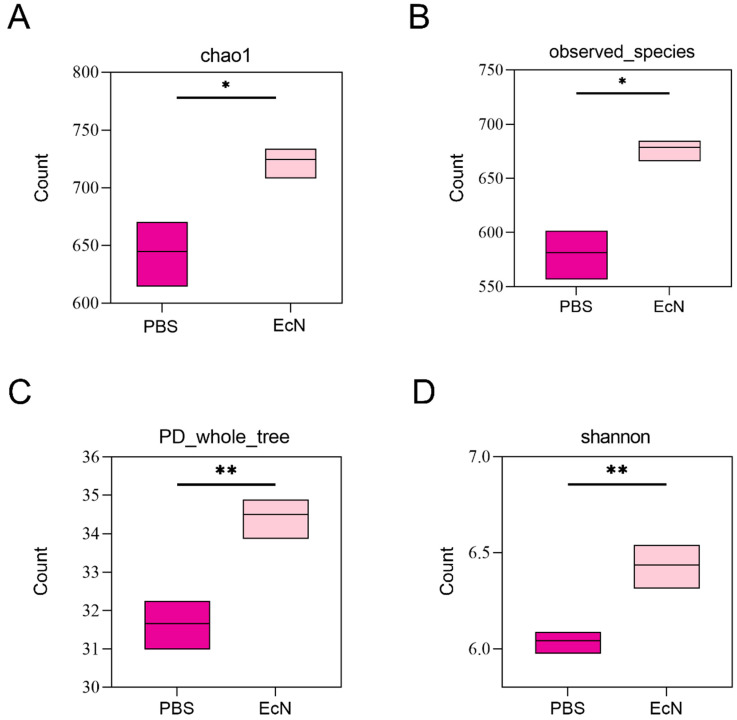
Analysis of alpha diversity in the two groups. Alpha diversity index of cecal microbiota adopt the Jaccard distance algorithm and use *t*-test to calculate the differences between groups. X-axis: experimental groups; Y-axis: specific alpha diversity index values. (**A**) Chao1 richness estimator. (**B**) Observed species index representation. (**C**) PD whole tree. (**D**) Shannon diversity index (* *p* < 0.05, ** *p* < 0.01). Box plots show median, quartiles, and whiskers extending to min/max values.

Shannon and dilution indices showed that the strain richness in the EcN group was higher than that in the control group, indicating that the microbial community diversity in the feces of the mice in the EcN group was higher than that in the control group. The Chao1 index (Figure 4A), observed species index (Figure 4B), PD whole tree index (Figure 4C) and Shannon-dilution index (Figure 4D) were calculated to test α diversity. These indices indicated that the richness of the control group was significantly higher than that of the EcN group.

#### 3.5.2. Beta Diversity

**Figure 5:**
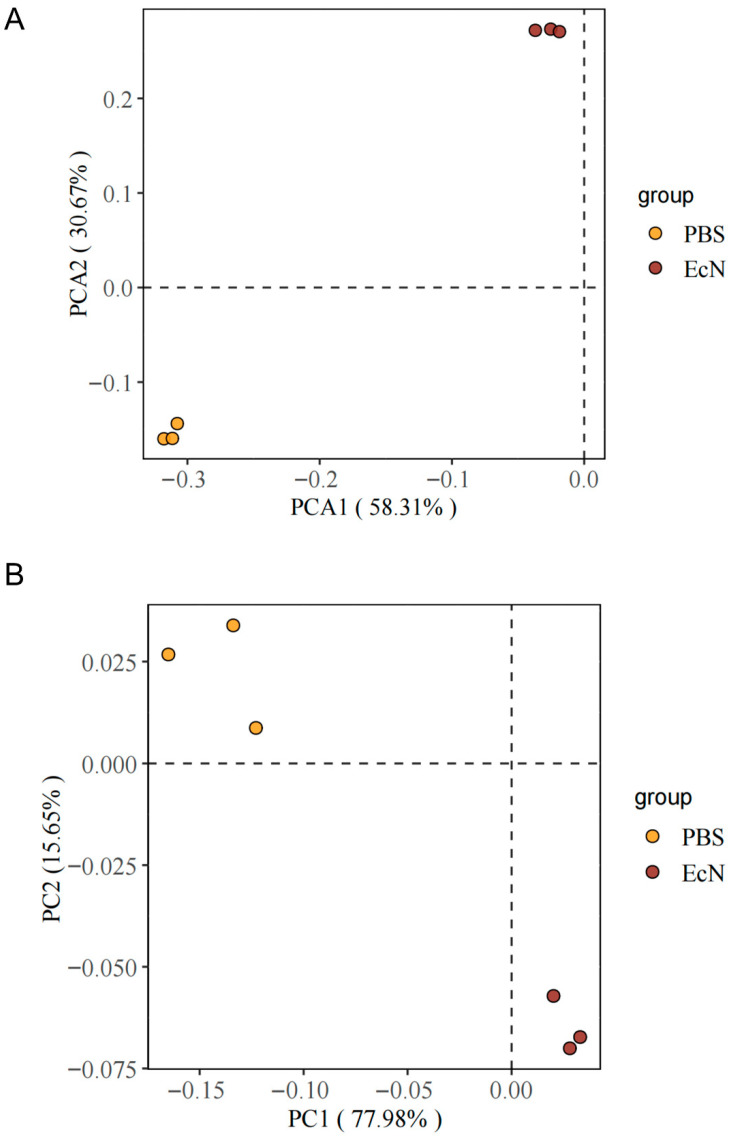
Principal coordinates analysis of the structure of the gut microbiota. Beta diversity also employs the Jaccard distance algorithm and uses *t*-test to calculate between-group differences. (**A**) Principal component analysis (PCA) based on operational taxonomic unit (OTU) abundance. (**B**) Principal coordinate analysis (PCoA) based on Bray–Curtis dissimilarity, showing beta diversity separation.

In this experiment, different color points represent different groups. With the development of infection, microbial flora was also affected. As shown in Figure 5, principal component analysis (PCA) and principal coordinate analysis (PCoA) indicated that there are remarkable variations in the microbial structure between the PBS and the EcN groups (Figure 5). Our data basically correspond to the α-diversity result of the Shannon–Wiener index. Overall, EcN played an important regulatory role in intestinal microbial communities in intracellular bacteria-infected mice, which is roughly corresponding to the α diversity results.

### 3.6. Taxa of Bacteria in Mice

**Figure 6:**
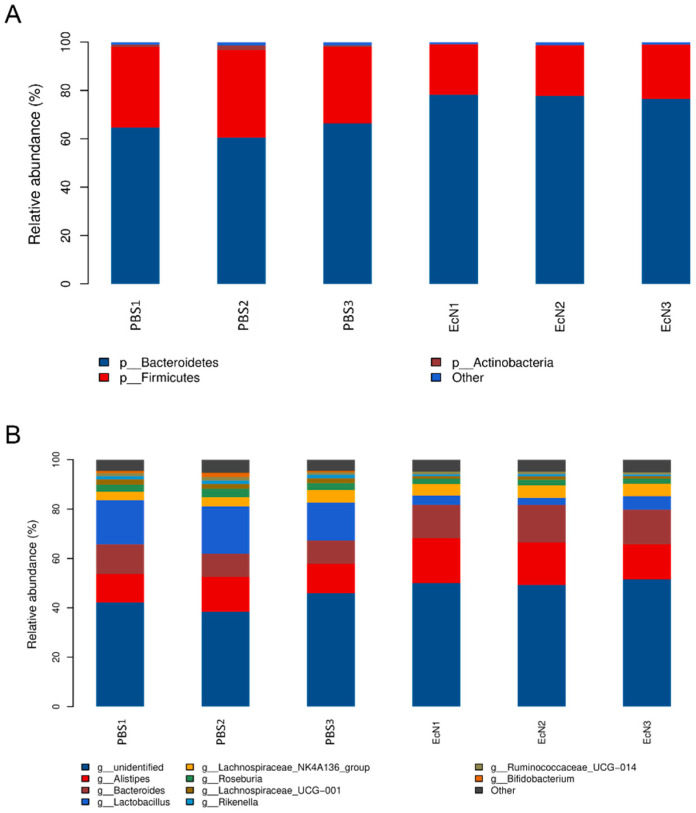
Gut microbiota composition analysis at the phylum (**A**) and genus (**B**) levels. Mice were orally administered PBS or *Escherichia coli* Nissle 1917 (Group EcN) for 7 days prior to *Salmonella typhimurium* infection. Fecal samples were collected and subjected to 16S rRNA gene sequencing to determine the relative abundance of bacterial taxa.

To further analyze the protection of EcN on *Salmonella typhimurium*-infected mice, we analyzed the microorganisms in the cecum at the phylum and genus level to determine the changes in microbial distribution. Analysis at the phylum level (Figure 6A): Three phyla were obtained, and *Bacteroidetes* was the dominant phylum. *Bacteroidetes* in group C comprised 63.91% and that in the EcN group comprised approximately 77.52%. *Firmicutes* accounted for 33.61% in group C and 21.36% in the EcN group. Compared with those in the EcN group, *Actinobacteria* and *Firmicutes* in group C increased, while *Bacteroidetes* decreased.

**Figure 7:**
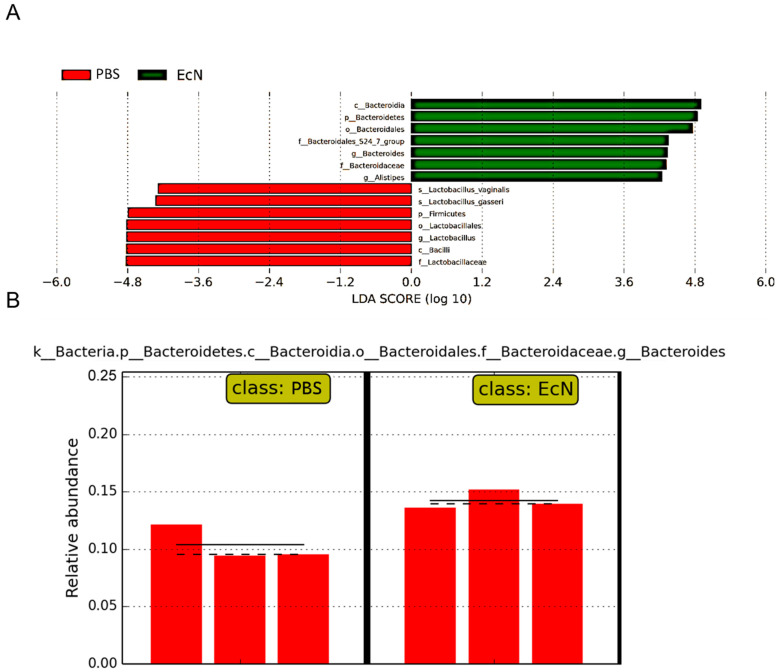
LDA scores obtained from the LEfSe analysis of the gut microbiota in different groups. (**A**) Cladogram illustrating the phylogenetic distribution of discriminative features, with LDA > 3.0 set as the significance threshold. (**B**) Effect size plot demonstrating that *Salmonella* challenge selectively reduced *Bacteroides* abundance, reflecting infection-induced dysbiosis. A co-occurrence network diagram was also performed to display the interactions among some major members belonging to *Proteobacteria* and *Firmicutes* (Figure 8A). Network analysis revealed a distinct clustering pattern between these two dominant genera, reflecting the dynamic interaction characteristics of the microbial community and suggesting that EcN treatment may modulate microbial ecological balance.

Genus-level analysis (Figure 6B): Eight genera were obtained, and *Alistipes*, *Lactobacillus* and *Bacteroider* were the dominant genera. *Bacteroides* and *Alistipes* in group C comprised 10.36% and 12.47%, respectively, and those in the EcN group were 14.25% and 16.50%, respectively, which were higher than those in group PBS. In contrast, *Lactobacillus* in the PBS group comprised 17.47%, while that in the EcN group was significantly decreased and was 4.12%. In addition, *Ruminococcaceae*, *Rikenella* and *Bifidobacterium* in group PBS were 1.24%, 1.38% and 1.01%, respectively. These three genera were unique to group C, suggesting that some genera were reduced or even disappeared after the mice received EcN. LefSe (LDA effect size) analysis showed that the levels of *Lactobacillus* in group PBS were remarkable higher than group EcN (Figure 7A). The amounts of *Bacteroides* in group EcN were obviously higher than those in group PBS (Figure 7B).

### 3.7. Metabolic Functional Analysis of Microbes

**Figure 8:**
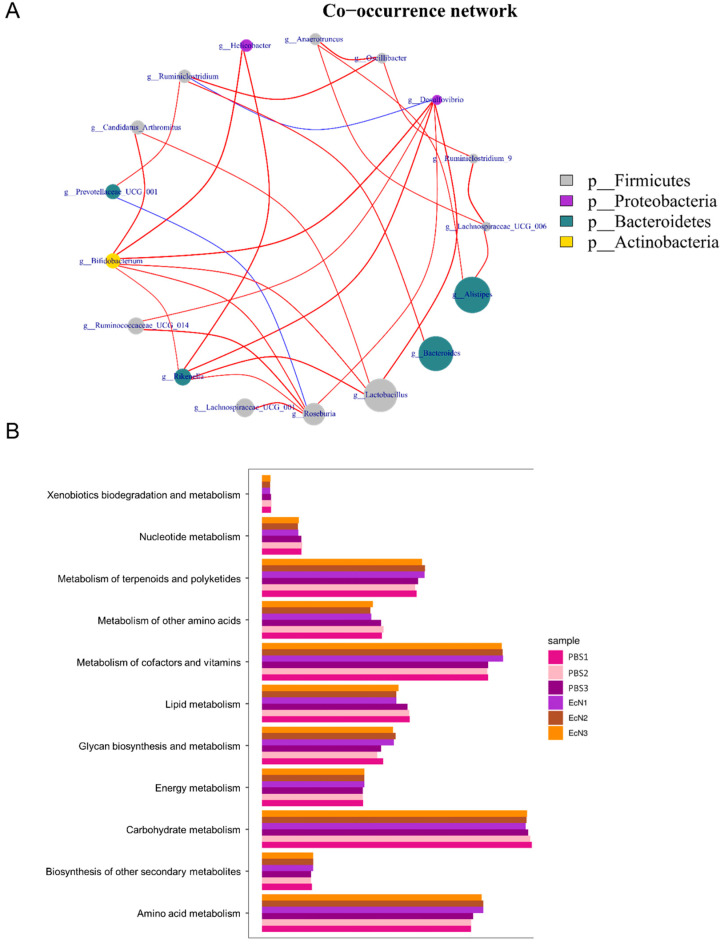
(**A**) Network analysis depicting inter-phylum interactions among four dominant bacterial phyla—Proteobacteria, Firmicutes, Bacteroidetes, and Actinobacteria—under EcN-mediated modulation. The red line represents positive correlation and blue line represents negative correlation. (**B**) Heatmap represents the PICRUSt2-predicted microbial metabolic functions across samples, anchored on KEGG ortholog abundance to illustrate hierarchical clustering of both specimens and functional modules.

A co-occurrence network diagram was also performed to display the interactions among some major members belonging to Proteobacteria and Firmicutes (Figure 8A). Based on the Kyoto Encyclopedia of Genes and Genomes (KEGG) pathway analysis, the metabolic function of microorganisms is shown in Figure 8B. The relative abundance of the KEGG pathways “Metabolism of terpenoids and polyketides”, “metabolism of cofactors and vitamins”, “carbohydrate metabolism and amino acid metabolism” were enriched (Figure 8B). The enrichment of these pathways enables the microbiota to synthesize antimicrobial/antiviral secondary metabolites, supply nutritional cofactors such as B vitamins, ferment short-chain fatty acids to maintain epithelial barriers, and convert amino acids into immune–regulatory signaling molecules, synergistically orchestrating host defense and microenvironmental homeostasis.

## 4. Discussion

In this study, we explored the protective effect of EcN on mice infected with *Salmonella typhimurium*. Flow cytometry was used to verify the effect of EcN on the immune system of the body. The results confirmed that EcN administration c increased the number of mature dendritic cells and promoted the immune response of the body. A previous study showed that EcN induced a dose-dependent upregulation of the expression of the cell maturation marker CD80/86 [30]. Our results were consistent with these findings. Dendritic cells, as the most powerful antigen-presenting cells, can directly interact with antigens in the intestine and cause an adaptive immune response. The CD80 and CD86 molecules expressed on the surface of DCs are not only signs of dendritic cell maturation but also important stimuli for activating T cells [31]. Therefore, the maturation of dendritic cells is extremely important for the body to cause an adaptive immune response [32]. The experiments have proven that EcN has immuno-stimulatory activity, which can induce the maturation of cells in the immune system and participate in the immune response.

Based on the weight loss rate in the experimental results, the mortality rate of the mice, and the amounts of bacteria both in the spleen and liver and the degree of pathological damage of related tissues, it could be clearly concluded that EcN can effectively reduce or resist the invasion of *Salmonella typhimurium* in mice. In the mice receiving EcN in advance, the weight loss was significantly reduced, the survival rate increased, and the number of bacteria in the tissues decreased. As shown by the sections, the pathological damage to the cecum, spleen and liver was less severe. These phenomena indicated that, when EcN exists in mice, the influence of *Salmonella typhimurium* on mice will be reduced.

The results of 16S high-throughput sequencing showed that EcN protected mice against harmful bacteria by regulating the distribution of microorganisms in the intestine. *Salmonella* sp. colonizes the intestine after entering the body, eventually reaching intestinal epithelial cells, damaging the barrier function of the intestine, leading to intestinal biological disorders and triggering further development of gastrointestinal diseases [33,34]. *Salmonella* sp. infection is often accompanied by other tissue damage, such as liver and spleen injury, manifested as inflammatory cell infiltration, severe congestion, hepatocyte apoptosis and oxidative damage [35]. EcN is a probiotic with a long history of application. On the one hand, it can send signals to host intestinal epithelial cells and stimulate cells to produce defensins, resulting in nonspecific defense mechanisms. On the other hand, it directly antagonizes other bacteria by producing bacteriocins and inhibits the growth of pathogenic bacteria in the intestine, adhesion to the intestine and further invasion, especially with sensitive strains of their close relatives [36].

The 16S rRNA sequencing results showed that *Lactobacillus* in the intestinal tract of the mice treated with EcN showed a significant decrease, while *Bifidobacterium* completely disappeared. We can classify some members of *Lactobacillus* and *Bifidobacterium* as probiotics; for example, *Lactobacillus amylovorus*, *Lactobacillus mucosae* [37,38] and PR4 (pig commensal strain of *Bifidobacterium choerinum*) [39] showed no obvious protective effect in *Salmonella*-infected piglets. In contrast, EcN was able to suppress clinical signs and histopathological changes. In previous studies, Souza et al. [6] found that the use of EcN to prevent IBD could improve tissue damage, improve mucosal integrity, and reduce the level of inflammatory factors. Jia-lu Shi et al. [40] used *Lactobacillus* to treat IBD and achieved similar effects.

Thus, these two probiotics have the same therapeutic effect. Coincidentally, in our previous experimental group, *Lactobacillus plantarum* was also used to protect mice with *Salmonella* infection, but the effect was not ideal. *E. coli* and *Salmonella* belong to *Enterobacteriaceae* of Proteus [41] and that *Lactobacillus* belongs to *Firmicutes*. However, EcN has been found to show resistance to inbreeding, so it has a better effect than *Lactobacillus* in the resistance to *Salmonella* [42]. However, some experiments have proven that *L. rhamnosus* can resist *Salmonella* infection [43]. Whether different genera of *Lactobacillus* in the intestine and their number will affect the body’s resistance to *Salmonella* is unclear.

For the increase in *Bacteroides* in the EcN group, it has been reported that the ratio of *Bacteroides* to total flora can be used as an indicator to determine IBD. Therefore, the number of *Bacteroides* is closely related to the inflammatory response in the intestinal tract. The increase in *Bacteroides* indicates that EcN has beneficially changed the distribution of advocated flora, further activated the innate immunity of the intestinal tract, and enabled the body to induce the inflammatory response of the intestinal tract [44]. The numbers of *Lactobacillus*, *Ruminococcaceae*, *Roseburia*, *Rikenella* and *Bifidobacterium* in the intestinal tract of the mice without EcN treatment were higher than those in the EcN treatment group, indicating that EcN reduced the abundance of intestinal colonies and adjusted the imbalance of intestinal microorganisms [45]. Their metabolites include acetic acid or other unique acid substances; for example, *Ruminococcaceae* produces formic acid [46], and *Rikenella* produces succinic acid [47], which increases the intestinal acidity, resulting in an acidic environment for microbial survival [48].

Through this experiment, we proved that EcN can play a protective role in mice infected with *Salmonella typhimurium*, which is also consistent with the view that EcN can antagonize closely related pathogenic bacteria. This study provided a reference for the future use of nonantibiotic preparations to treat bacterial diseases and demonstrated the antibacterial potential of probiotics. These results suggested that we can use the interaction of multiple bacteria to regulate the distribution of microbial flora [49]. The development of probiotics will become a promising research direction in the future when antibiotic use becomes increasingly strict.

## 5. Conclusions

The obtained results confirmed that EcN administration increased the number of mature dendritic cells. Furthermore, it can effectively reduce or resist the invasion of *Salmonella typhimurium* in mice represented by decreased weight loss, mortality rates, the bacterial load in the internal organs including the spleen and liver, and the degree of pathological damage of related tissues. It was clearly obvious that EcN protected the tested mice against the harmful effect of bacteria via not only regulating the distribution of microorganisms in the intestine, but also through reducing the abundance of intestinal colonies and adjusting the imbalance of intestinal microorganisms. Such optimistic results encourage application of EcN as a prophylactic approach and intervention with an unusual treatment to improve the animal health of multiple target species. Also, the obtained results motivate evaluating the protection level of EcN against different pathogenic organisms.

## Data Availability

The DNA sequences generated and analyzed during the current study are available in the NCBI Sequence Read Archive database (accession: SRP328586).
